# BCL2i-Based Therapies and Emerging Resistance in Chronic Lymphocytic Leukemia

**DOI:** 10.3390/cells13221922

**Published:** 2024-11-20

**Authors:** Wing Fai Li, Eleftheria Atalla, Jiaxin Dong, Marina Konopleva

**Affiliations:** 1 Department of Internal Medicine, Jacobi Medical Center, Bronx, NY 10461, USA; liw4@nychhc.org; 2 Department of Hematology and Oncology, The University of Texas at San Antonio, San Antonio, TX 78249, USA; atallae@uthscsa.edu; 3 Department of Oncology, Albert Einstein College of Medicine, Bronx, NY 10461, USA; jiaxin.dong@einsteinmed.edu

**Keywords:** BCL2i, venetoclax, drug resistance, clinical trials, chronic lymphocytic leukemia

## Abstract

Overexpression of the anti-apoptotic protein BCL-2 is a key factor in the pathogenesis of chronic lymphocytic leukemia (CLL) and is associated with poor clinical outcomes. Therapeutic activation of apoptosis in cancer cells using the BCL-2 inhibitor (BCL2i) venetoclax has shown remarkable efficacy in clinical trials, both as monotherapy and combination regimens. However, patients with CLL experience a highly variable clinical course, facing significant challenges in advanced stages due to disease relapse and the emergence of resistant clones. Resistance mechanisms include acquired *BCL-2* mutations, alteration of pro-apoptotic and anti-apoptotic proteins, metabolic reprogramming, epigenetic changes, and aberrant signaling pathways. To address this complex disease and improve progression-free survival, strategies targeting multiple signaling pathways and mechanisms have been explored. Randomized clinical trials of venetoclax in combination with Bruton tyrosine kinase (BTK) inhibitors or CD20 monoclonal antibodies have significantly outperformed traditional chemoimmunotherapy in both treatment-naïve and relapsed patients, achieving undetectable minimal residual disease (uMRD) and durable remissions. This review explores the intricate balance between BCL-2 family proteins and their role in the intrinsic apoptosis pathway, discusses venetoclax resistance mechanisms, and highlights the evolving role of venetoclax and other BCL2i-based combination therapies in CLL treatment.

## 1. Introduction

Apoptosis or programmed cell death, initially discovered as a developmental process of the nematode *Caenorhabditis elegans*, has enriched our understanding of physiology and diseases [1]. The balance between life and death is delicate. In normal cellular function, a cell undergoes growth, sustains itself, enters senescence, and ultimately dies, clearing the way for the formation of new cells. This orchestrated process ensures proper cellular turnover, homeostasis, and supports overall development. However, in many cancers, such as chronic lymphocytic leukemia (CLL), this intricate balance is disrupted, allowing for malignant cells to proliferate and adapt.

CLL is the most prevalent leukemia among adults and accounts for 1 in 3 new cases in the United States, with a median age at diagnosis between 67 and 72 years [2,3]. This indolent hematologic malignancy is marked by clonal expansion of mature B lymphocytes that are CD5+ CD19+ CD23+ in the blood, lymph nodes, and bone marrow [4]. The hallmark of this disease is the highly variable clinical course due to the genetic and epigenetic heterogeneity [5]. While some patients may survive for years without requiring immediate treatment, others experience rapid disease progression within months [4,6]. Despite the availability of various treatments that can induce temporary remissions, continuous therapy often leads to the development of resistant cellular clones and eventual disease relapse, rendering CLL largely incurable in its advanced stages [7,8].

Over the 15 years, notable advancements in the treatment landscape of CLL have been made, particularly with the introduction of targeted therapy against B-cell lymphoma 2 (BCL-2) within the apoptosis pathway and non-receptor tyrosine kinase Bruton tyrosine kinase (BTK) [9,10]. Venetoclax, a specific BCL-2 antagonist, has transformed the treatment of CLL. Venetoclax-based regimens have increased the progression-free survival (PFS) and overall survival rate in newly diagnosed and relapsed/refractory CLL patients. This review aims to explore the intricate balance and interplay between BCL-2 family protein and the critical role of BCL-2 in the intrinsic pathway of the apoptosis cascade. We will also explore the mechanisms behind venetoclax resistance and clinical management of CLL. By analyzing past and ongoing clinical trials, we hope to provide insights into the efficacy and potential future applications of venetoclax and other BCL2i in CLL treatment.

## 2. Apoptosis Pathway

**Figure 1:**
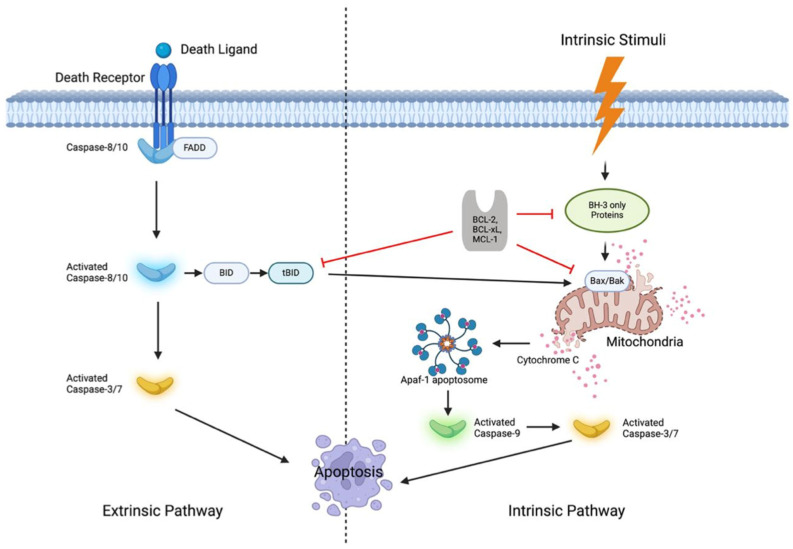
This Figure illustrates the intrinsic and extrinsic pathways of apoptosis, emphasizing the role of BCL-2 family proteins in regulating cell death. The extrinsic pathway is triggered by death ligands binding to their receptors, activating FADD and caspase-8, which cleaves BID into its active form, tBID. tBID translocates to the mitochondria and engages the intrinsic pathway, where the BCL-2 family—comprising anti-apoptotic proteins (BCL-2, BCL-xL, MCL-1), pro-apoptotic effectors (BAX, BAK), and BH3-only proteins (BID, PUMA)—regulates mitochondrial outer-membrane permeabilization (MOMP). Upon cellular stress, BH3-only proteins inhibit anti-apoptotic BCL-2 members, freeing BAX and BAK to form oligomers that create pores in the outer mitochondrial membrane, releasing cytochrome c to activate Apaf-1 and caspase-9, leading to apoptosis. Therapeutic BH3 mimetics target this balance, inducing apoptosis in cells dependent on BCL-2, BCL-xL, or MCL-1 for survival. Apaf-1: Apoptotic Peptidase Activating Factor 1; BCL-2: B-Cell Lymphoma 2; BCL-xL: B-Cell Lymphoma Extra Large; FADD: Fas-Associated Death Domain; BID: BH3 Interacting Domain Death Agonist; tBID: Truncated BID; MCL-1: Myeloid Cell Leukemia 1; BH3: BCL-2 Homology 3; Bax: BCL-2-Associated X Protein; Bak: BCL-2 Antagonist Killer.

Apoptosis is a conserved, complex process involving the extrinsic (death-receptor-mediated) and intrinsic (mitochondrial-mediated) pathways [11] (Figure 1). The extrinsic pathway is initiated by external ligands such as FasL, TRAIL, and TNFα or by the release of granzyme B and perforin from cytotoxic lymphocytes. These signals activate death receptors on the cell surface, setting off a series of molecular events that result in programmed cell death [12]. Upon activation, the death receptors undergo trimerization and recruit adapter proteins such as FADD (Fas-associated death domain) and TRADD (TNFR-associated death domain), forming the death-inducing signaling complex (DISC) [13]. This complex serves as the platform for the activation of caspase-8, which then amplifies and propagates the apoptotic signal through sequential effector caspases like caspase-3, -6, and -7 [14,15].

The intrinsic pathway, in contrast, is largely centered on the regulation of the BCL-2 family. BCL-2 was first reported as an oncogene in the translocation (14;18) in patients with follicular lymphoma [16]. The BCL-2 proteins include pro-survival proteins such as BCL-2, BCL-XL, and MCL-1 [17]; pro-apoptotic effector proteins: BAX, BAK, and BOK [18]; and BH3-only proteins: BIM, PUMA, NOXA, BAD, BID, and others [17]. These proteins contain four highly conserved BCL-2 homology (BH) domains— BH1, BH2, BH3, and BH4—which are crucial for the functional roles of BCL-2 family molecules [19]. Pro-survival members share sequence homology at BH1 through BH4, while pro-apoptotic proteins, BAX, BAK, and BOK, share sequence homology at BH1, BH2, and BH3, except for BH4 [20]. All BH3-only proteins share only the BCL-2 homology region 3 (BH3-only) and are intrinsically disordered, except for BID [21]. Structurally, the BCL-2 family protein has a conserved globular core with a hydrophobic groove which allows for BCL-2 proteins to exert their versatile function mainly through protein–protein interactions [19]. These interactions occur mainly within the membrane as described in the “embedded together” model [22].

Under normal conditions, when a cell is subjected to stress, such as hypoxia or nutrient insufficiency, BH3-only protein levels rise, which neutralize and inhibit pro-survival BCL-2 proteins. This inhibition frees BAX and BAK, allowing them to form dimers and permeabilize the mitochondrial outer membrane [18]. This permeabilization results in the release of cytochrome c from the mitochondria into the cytoplasm, where it binds to apoptotic protease activating factor 1 (APAF-1). This interaction leads to the oligomerization of APAF-1 and the formation of the apoptosome [23]. The apoptosome then activates downstream caspases, resulting in cleavage of structural proteins, DNA repair enzymes, and other essential molecules [24]. Along with cytochrome c, other mitochondrial proteins, such as SMAC/DIABLO and HTRA2 are also released into the cytoplasm [25]. These mitochondrial proteins further enhance apoptosis by inhibiting XIAP, a caspase inhibitor, ensuring efficient cell death [25].

## 3. The Life/Death Switch by BCL-2 Family Proteins

The tug-of-war between pro-survival and pro-apoptotic factors determines the trigger of the death cascade via MOMP and permeabilization of the outer mitochondrial membrane [26]. The regulation of cellular survival and death by BCL-2 proteins is highly intricate and far more nuanced than previously thought. A central aspect of this regulation involves BH3-only proteins, which act as activators by binding to anti-apoptotic proteins. This binding causes pro-apoptotic BCL-2 proteins to undergo dramatic conformational changes, exposing the BH3 domain and allowing for interactions with the BAX groove. This triggers the separation of the core domain and latch domains of BCL-2, facilitating BAX dimerization and the subsequent permeabilization of the mitochondrial outer membrane [27,28]. Alternatively, BH3-only proteins act as sensitizers by the BH3-binding groove of anti-apoptotic proteins and displace BAX/BAK [28].

One common mechanism for evading apoptosis in cancers is the overexpression of BCL-2 proteins, observed in various cancers, such as prostate, colorectal, lung, gastric, breast, lymphoma, and leukemia [29,30,31,32,33]. The interplay between BCL-2 signaling and other abnormal metabolic and growth pathways is well-documented in cancer biology. For example, overexpression of oncogene c-MYC, found in nearly 40% of cancers, drives unchecked cell-cycle progression and autonomous tumor growth, increasing sensitivity to apoptosis, largely through p53 [34,35]. Conversely, inactivation or downregulation of pro-apoptotic proteins such as BID, BIM, BOK, and PUMA contributes to tumorigenesis [36,37].

Post-translational modifications, including phosphorylation and epigenetic regulation, also impact the binary switch between life and death in cancer cells. For example, hypermethylation of BCL-2, BAX, BAK, and PUMA promoter are implicated in prostate cancer, multiple myeloma, and lymphoma, highlighting the role of epigenetic alterations in apoptosis resistance [38,39,40]. Dephosphorylation of specific MAP kinase sites (such as Ser87) within the BCL-2 protein facilitates its ubiquitin-dependent degradation via the proteasome. MAP kinases, particularly ERK1/2, stabilize BCL-2 through phosphorylation, protecting against apoptosis. Reduced ERK activity leads to BCL-2 dephosphorylation and subsequent degradation [41]. Additionally, hypoxia reduces the levels of nearly all pro-apoptotic BCL-2 family proteins, including NOXA and BAD, in in vitro settings through both p53-dependent and p53-independent pathways [42].

## 4. Activation of Apoptosis with BCL-2 Inhibitors

Leukemic CLL cells, in particular, are highly BCL-2-dependent for their growth and survival, which is achieved through the overexpression of this anti-apoptotic protein [43,44,45]. In approximately 10% of B-cell CLL, the BCL-2 gene is translocated into the immunoglobulin (Ig) loci, leading to abnormally high levels of BCL-2 expression [46]. This overexpression enables malignant B cells to evade apoptosis, the programmed cell death that typically eliminates damaged or unnecessary cells, thereby promoting their uncontrolled proliferation through BCR-mediated signaling [47]. As a result, CLL cells with elevated BCL-2 expression exhibit prolonged survival and delayed DNA degradation in response to cellular stress compared to CLL cells with lower BCL-2 protein levels [48]. Moreover, BCL-2 overexpression is strongly associated with adverse outcomes in CLL patients [49].

The identification of BCL-2 as a key driver of cancer pathogenesis has paved the way for the development of BCL-2 inhibitors. However, designing effective inhibitors has been challenging due to the dynamic nature of protein–protein interactions involving a shallow, hydrophobic binding groove. A major breakthrough in BCL-2 inhibitor development came with nuclear magnetic resonance (NMR)-based screening, which identified small molecules capable of blocking the hydrophobic BH3-binding domain of BCL-family protein. ABT-737, a small molecule inhibitor, was shown to be capable of binding to BCL-2, BCL-XL, and BCL-w with high affinity (Ki ≤ 1 nM). By binding to these anti-apoptotic proteins, ABT-737 disrupts their interactions with pro-apoptotic BH3-only family members, enhancing the death signal and leading to mitochondrial outer membrane permeabilization [50].

Navitoclax (ABT-263), a second-generation inhibitor, improved upon ABT-737 by being a potent, orally bioavailable BCL-2 and BCL-XL inhibitor, offering greater clinical utility as a monotherapy [51]. A phase-I trial with navitoclax in patients with relapsed/refractory CLL reported progression-free survival of 25 months, with 31% of patients achieving a partial response, and the majority of patients had more than half reduction in peripheral lymphocyte count [52]. However, navitoclax’s major dose-limiting toxicity is drug-induced thrombocytopenia [52], with 38.5% of patients in a subsequent phase 2a study developing thrombocytopenia [53]. Mechanistic study reveals that navitoclax’s dose-dependent thrombocytopenia is related to BCL-XL inhibition, which is toxic to the survival of the mature circulating platelets [54].

Given the promising future of BCL-2 inhibition, further chemical re-engineering produced venetoclax (ABT-199), a highly selective BCL-2 inhibitor [45]. As a BH3 domain mimetic, venetoclax binds directly to BCL-2 and replaces BIM or other pro-apoptotic proteins bonds to BCL-2. This reactivates the apoptotic pathway and promotes oligomerization of BAX and BAK, thus selectively inducing death in CLL cells [55]. Preliminary clinical data have highlighted venetoclax’s exceptional efficacy, particularly in patients with relapsed or refractory CLL, including those with high-risk cytogenetic markers, such as del(17p) [56]. Venetoclax monotherapy achieved rapid reductions in disease burden, with overall response rates of approximately 80% and complete response rates ranging from 6% to 20%. As a result of these promising findings, venetoclax received full FDA approval for use as a monotherapy in CLL in 2016 [56].

## 5. Mechanism of Resistance of Venetoclax

**Figure 2:**
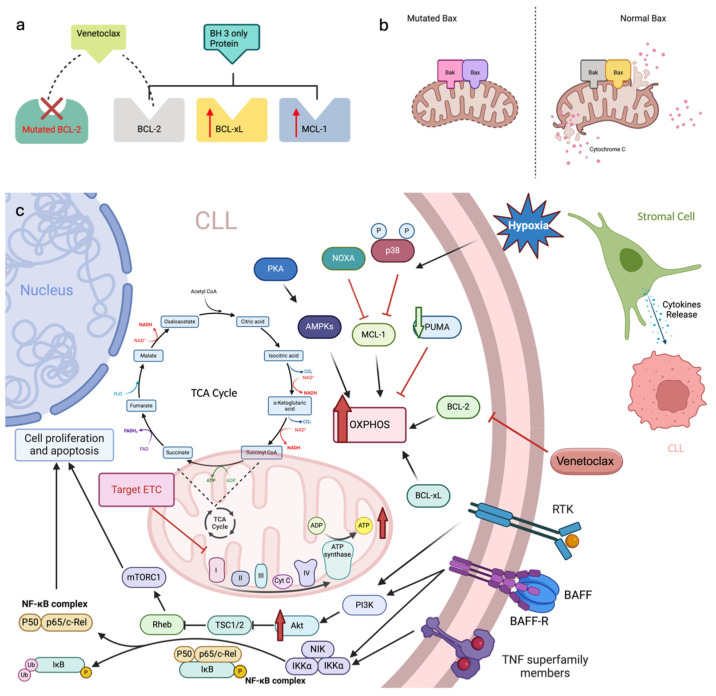
Mechanisms of resistance to venetoclax in leukemic cells through multiple pathways: acquired *BCL-2* mutations, mutated BAX, metabolic, epigenetic, and signaling reprogramming. (**a**) Acquired *BCL-2* mutations: Mutations in *BCL-2* reduce venetoclax’s binding affinity, allowing for cell survival. Compensatory upregulation of BCL-xL and MCL-1 further diminishes venetoclax’s effectiveness. (**b**) Mutated *BAX*: Mutations in *BAX* prevent pore formation and cytochrome c release, thereby blocking mitochondrial apoptosis even when BCL-2 is inhibited. (**c**) Metabolic and signaling reprogramming: Cancer cells adapt by increasing dependency on OXPHOS involves enhanced activity of the ETC, including complexes I-IV and Cyt C, activating pro-survival pathways such as PI3K/AKT/mTOR and NF-κB, and undergoing epigenetic modifications. These alterations shift dependency from BCL-2 to MCL-1 or BCL-xL. BAFF further supports pro-survival signaling through NF-κB, while NOXA, a BH3-only protein, competes for binding sites on anti-apoptotic proteins like MCL-1, influencing apoptotic sensitivity. These adaptations are influenced by the tumor microenvironment, including stromal interactions and cytokine signaling, which collectively support cell survival and venetoclax resistance. NAD: Nicotinamide Adenine Dinucleotide; NADH: Reduced Nicotinamide Adenine Dinucleotide; TCA: Tricarboxylic Acid; CO₂: Carbon Dioxide; CoA: Coenzyme A; Cyt C: Cytochrome C; ADP: Adenosine Diphosphate; ATP: Adenosine Triphosphate; PKA: Protein Kinase A; AMPKs: AMP-Activated Protein Kinases; PUMA: p53 Upregulated Modulator of Apoptosis; p38: p38 Mitogen-Activated Protein Kinase; RTK: Receptor Tyrosine Kinase; CLL: Chronic Lymphocytic Leukemia; TNF: Tumor Necrosis Factor; PI3K: Phosphoinositide 3-Kinase; Akt: Protein Kinase B; TSC1/2: Tuberous Sclerosis Complex 1/2; Rheb: Ras Homolog Enriched in Brain; mTORC1: Mechanistic Target of Rapamycin Complex 1; ETC: Electron Transport Chain; NIK: NF-κB-Inducing Kinase; IKKα: IκB Kinase α; P50: Nuclear Factor NF-κB P50 Subunit; p65/c-Rel: NF-κB p65/c-Rel Subunit; IκB: Inhibitor of κB; Ub: Ubiquitin; P: Phosphate Group; NF-κB: Nuclear Factor κ-Light-Chain-Enhancer of Activated B Cells; OXPHOS: Oxidative Phosphorylation; NOXA: Phorbol-12-Myristate-13-Acetate-Induced Protein 1 (PMAIP1); BAFF: B-cell Activating Factor.

Venetoclax has revolutionized the treatment of CLL, yet relapses remain common after initial remission. Multiple resistance mechanisms have emerged, including genomic mutations, epigenetic changes, alternative survival pathways, metabolic reprogramming, and modifications in the apoptotic machinery, all of which challenge effective CLL management (Figure 2).

### 5.1. Genomic Mutations and Epigenetic Changes

Venetoclax specifically targets BCL-2, so it is no surprise that any alteration to the structure of the target could potentially impact the drug’s effectiveness. Studies have identified de novo mutations in venetoclax-treated cells, including the G101V mutation, where glycine at position 101 is substituted by valine, impairing the BH3-binding domain by approximately 180-fold [57]. This mutation was first detected in patients with initial treatment responses but relapsed after 19 to 42 months. Notably, this mutation does not affect BCL-2’s interaction with other pro-apoptotic proteins, enabling malignant cells to evade apoptosis [58]. Structural analyses show that G101V may exert downstream knock-on effects on E152, a residue adjacent to V101 [59]. The D103Y mutation is another resistance mutation discovered through next-generation sequencing in a patient’s peripheral blood samples after 39 months of venetoclax treatment [60]. Like the G101V mutation, D103Y disrupts the BH3-binding P4 pocket of the BCL-2 protein. Aspartic acid (D) at position 103, situated near the BH3-binding groove targeted by venetoclax, is replaced by tyrosine (Y), leading to conformational changes that reduce the drug’s binding efficacy. Additionally, sequencing data from eight CLL patients who developed resistance to venetoclax revealed recurrent mutations in *BTG1* (B cell translocation gene 1), homozygous deletions affecting cyclin-dependent kinase inhibitor 2A/B (*CDKN2A/B*), mutation in *BRAF*, and a high-level focal amplification of *CD274 (PD-L1)* [61]. Particularly, BTG1 has been shown to be a crucial regulator of B cells’ adaptive immune response and primed for DLBCL-like lymphomas transformation [62].

Although prolonged treatment induces genomic mutations that confer resistance to venetoclax, the role of epigenetic modification in venetoclax resistance remains poorly understood. A recent study using B-cell lymphoma cell lines and primary CLL cells demonstrated that CpG island methylation within the PUMA promoter after venetoclax treatment downregulates PUMA expression at both the transcript and protein levels [58]. This hypermethylation alters interactions among apoptotic proteins, shifting the survival dependence of resistant tumor cells from BCL-2 to MCL-1, which decreases venetoclax sensitivity [63]. In CLL patients, DNA methylation levels were found to increase by 10–30%, underscoring the role of PUMA promoter methylation in the development of resistance [58].

### 5.2. Alteration in Anti-Apoptotic and Pro-Apoptotic Proteins

Resistance to venetoclax may develop through increased expression of pro-survival proteins, such as MCL-1 and BCL-XL, which can bind and neutralize pro-apoptotic proteins like BIM, enabling cancer cells to evade apoptosis despite venetoclax therapy. Haselager et al. demonstrated that resistance driven by elevated levels of pro-survival BCL-2 family members, particularly BCL-2, followed by BCL-XL, MCL-1, and BFL-1. Notably, CLL cells with elevated levels of BCL-XL and MCL-1 are more resistant to venetoclax, with BCL-XL exerting a more dominant influence on this resistance [64], which may be overcome by introducing inhibitors targeting these two proteins, achieving synergy with venetoclax. These molecular alterations disrupt the balance between pro-apoptotic and anti-apoptotic signals, favoring cell survival over apoptosis.

Additionally, BAX and BAK are critical for the execution of apoptosis by inducing mitochondrial outer membrane permeabilization. Loss of function mutations in *BAX* were identified in relapsed or refractory (R/R) high-risk CLL cases [58] (Figure 2b). In cellular assays, C-terminal BAX mutants disrupted the localization of BAX to the outer mitochondrial membrane, leading to resistance against venetoclax-induced apoptosis. Single-cell sequencing showed a clonal co-occurrence of *BAX* mutations with *DNMT3A* or *ASXL1* mutations, indicating a lineage-specific adaptation to venetoclax [65].

### 5.3. Metabolic Reprogramming

The tumor microenvironment plays a role in venetoclax resistance by offering external survival signals through metabolic changes, interactions with stromal cells, and survival-promoting cytokines, which counteract the drug’s pro-apoptotic effects. Early studies revealed three distinct functional states in apoptosis: unprimed, primed for death, and dead, with only “primed for death” cells being sensitive to apoptotic signals, driven by pro-apoptotic BCL-2 proteins [66,67]. In the lymph node microenvironment, CLL cells exhibit elevated levels of anti-apoptotic proteins BCL-XL and MCL-1, while the expression of the pro-apoptotic protein NOXA is suppressed. This imbalance reduces the cells’ sensitivity to venetoclax and has been linked to increased resistance to treatment [64,68].

In hypoxic conditions that resemble the lymph node microenvironment, activation of p38 MAPK downregulates MCL-1, thereby increasing the effectiveness of BH3 mimetics [69]. This p38-MCL-1 axis has been identified as a critical determinant of CLL cell sensitivity to BH3 mimetics, including venetoclax. However, p38 MAPK inhibition can restore MCL-1 expression, thus reducing the BH3 mimetics effectiveness in hypoxic conditions.

Additionally, mitochondrial metabolic reprogramming in CLL cells often leads to increased reliance on oxidative phosphorylation (OXPHOS), further diminishing venetoclax sensitivity [70]. This metabolic shift lowers BCL-2 dependency, allowing for venetoclax resistance. Additionally, the loss of PUMA, associated with metabolic reprogramming, enhances OXPHOS and ATP production, mirroring the metabolic phenotype seen in venetoclax-resistant cells [58]. Inhibiting the electron transport chain has shown potential in overcoming venetoclax resistance by lowering BCL-XL and MCL-1 levels at the mitochondrial OXPHOS site [71]. Furthermore, increased AMPK/PKA signaling and altered energy metabolism pathways contribute to venetoclax resistance [72] (Figure 2c).

### 5.4. Aberrant Signaling Pathways

Multiple signaling pathways contribute to venetoclax resistance in CLL, notably the PI3K/AKT/mTOR and NF-κB pathways, each with distinct roles in promoting cell survival and proliferation. The PI3K/AKT/mTOR pathway becomes activated with prolonged venetoclax exposure, leading to increased levels of MCL-1 and BCL-XL, which sequester the pro-apoptotic protein BIM and thus promote resistance. Choudhary et al. demonstrated that venetoclax-resistant cell lines exhibit significant AKT activation and MCL-1 upregulation, which could be countered by PI3K, AKT, and mTOR inhibitors, underscoring the potential of dual inhibition strategies for overcoming resistance lymph node microenvironment [73].

In contrast, NF-κB signaling, frequently activated in the lymph node microenvironment via CD40, enhances BCL-XL expression through both canonical and non-canonical pathways [74]. Targeting non-canonical NF-κB signaling can reduce BCL-XL expression, increasing CLL cell sensitivity to venetoclax [75]. Single-cell multiomics data also indicate that MCL1 may be a direct transcriptional target of NF-κB and that NF-κB activation is a marker of relapse in patients undergoing venetoclax therapy [76]. Additionally, studies suggest that ibrutinib enhances venetoclax efficacy by reducing BCL-XL and MCL-1 levels as it relocates CLL cells from the lymph nodes to peripheral blood, where survival signals are less abundant [64,75]. Recent data have shed light on the importance of BAFF, a B cell survival cytokine from the TNF family. It is shown to activate non-canonical NF-κB signaling and is crucial for B cell survival and maturation [77,78]. This role of BAFF is evident not only in BAFF-overexpressing mice, which develop autoimmune-like symptoms and B-cell lymphoproliferative disorders, but also in CLL patients treated with venetoclax or the anti-CD20 antibody Obinutuzumab showing markedly elevated levels of BAFF, leading to sustained pro-survival protein expression in leukemic cells over time [79,80]. A phase II trial of belimumab, an anti-BAFF monoclonal antibody, in combination with rituximab/venetoclax is currently being investigated in patients with refractory or relapsed CLL (NCT05069051).

Other mechanisms contributing to venetoclax resistance include ROR1 signaling, which enhances BCL-XL expression via the ERK1/2 and NF-κB pathways [81], and the JAK1/2-STAT3 pathway, which upregulates BCL-2, MCL-1, and BCL-XL in response to IFN-γ, further strengthening resistance [82]. Inhibition of NF-κB-inducing kinase (NIK) disrupts NF-κB- and STAT-signaling crosstalk, resensitizing CLL cells to venetoclax [83]. Table 1 summarizes key genetic mutations and alterations associated with resistance to venetoclax-based therapies in CLL. It includes the affected genes, approximate prevalence rates based on patient studies, and the role of each mutation in contributing to resistance.

The prevalence and clinical significance of different venetoclax resistance mechanisms in CLL underscore the complexity of treatment-resistant disease. Clonal evolution, as demonstrated in recent studies, emerges as a primary driver of resistance, with therapeutic pressure fostering the selection of new, resistant subclones. This evolutionary mechanism is both prevalent and clinically significant, particularly in high-risk or relapsed CLL, as it limits treatment efficacy and duration [87,88]. Additionally, *BCL-2* mutations, notably the G101V mutation, are identified as a potent resistance mechanism in some cases, directly impairing venetoclax binding and leading to disease progression; however, their occurrence appears limited across patient populations, suggesting clinical relevance when present but not widespread prevalence [57]. Furthermore, recurrent genetic alterations, such as *BTG1* mutations and *CDKN2A/B* deletions, observed in certain patients provide alternative resistance pathways, indicating that while these mutations are less common, they are clinically significant and potentially targetable in subsets of patients [86]. The CLL microenvironment also plays a critical role in promoting venetoclax resistance, with survival signals from the lymphoid niche supporting resistant clones. High-level *CD274* (*PD-L1*) amplification in one case highlights immune evasion as another resistance pathway, reinforcing the need for combination therapies that target both intrinsic and extrinsic resistance factors [61]. Together, resistance to venetoclax is complex, driven by clonal evolution and microenvironmental influences, supporting the necessity for personalized treatment approaches in venetoclax-resistant CLL.

## 6. Why Is Combining Therapy a Good Strategy?

Previous studies of venetoclax monotherapy primarily showed partial therapeutic responses, with remissions featuring undetectable minimal residual disease (uMRD) in the bone marrow being rare. These outcomes underscore the potential advantages of combination therapy in achieving deeper and more sustained responses in CLL patients [89]. Numerous venetoclax-based therapies have shown synergistic effects, offering deeper and more durable remissions. Some of the prominent venetoclax-based clinical trials are highlighted in Table 2.

CD20 receptors, which are closely associated with B-cell receptors, are highly effective targets for monoclonal antibody therapies. Anti-CD20 monoclonal antibodies, including rituximab, obinutuzumab, and ublituximab, have shown significant efficacy when combined with venetoclax in treating CLL. Early preclinical studies have demonstrated substantial cytotoxic synergy between venetoclax and anti-CD20 antibodies, as observed in the combinations of venetoclax with rituximab or obinutuzumab. These combinations not only counteract venetoclax resistance but also further enhance apoptosis in CLL cells by downregulating MCL-1 [96,101,102]. Clinical trials have reinforced these findings; for instance, in a phase Ib dose-escalation trial (NCT01682616) involving venetoclax and rituximab (VenR), it reported deep, durable responses, with 74% of these responses sustained for over five years. Notably, the VenR regimen demonstrated a progression-free survival rate of 56% and an overall survival rate of 86% at a median follow-up of 5.3 years. Re-treatment with VenR post-disease progression yielded favorable responses, highlighting its potential as an effective therapeutic strategy [103].

In 2019, Jain et al. published the results from a phase II study (NCT02756897) evaluating the combination of venetoclax and the BTK inhibitor ibrutinib in 80 previously untreated, high-risk, older patients with CLL. After 12 cycles of treatment, 88% of patients achieved CR/CRi, and 61% attained remission with uMRD [91]. Similar outcomes were observed in the CLARITY Study, which combined ibrutinib with venetoclax in 54 patients with relapsed or refractory CLL. In this cohort, 53% of patients achieved MRD negativity in blood, 36% in the bone marrow, and 51% reached complete remission, with the regimen demonstrating favorable tolerability [92]. A parallel phase II trial (NCT03045328) at Stanford and City of Hope reported a 91% ORR, 55% CR, and MRD negativity rates of 65% and 75% at one and two years, respectively, after treatment with ibrutinib with venetoclax [104]. Similarly, for treatment-naive CLL patients with high-risk features, such as del(*17p*) or *TP53* mutation, the combination of ibrutinib and venetoclax achieved bone marrow MRD negativity in 56% of patients, increasing to 66% after 24 cycles (NCT02756897). Across all high-risk subgroups, 75% of patients achieving bone marrow MRD negativity, with durable responses. The 3-year PFS and OS rates were 93% and 96%, respectively, and the safety profile was manageable [91,105].

The GLOW trial (NCT03462719) is the subsequent randomized study that evaluated the ibrutinib and venetoclax (I+V) combination in patients with CLL [97]. It specifically assessed the efficacy and safety of a fixed-duration I+V regimen compared to chlorambucil plus obinutuzumab in older or comorbid, treatment-naïve CLL patients. I+V demonstrated a significantly longer PFS than the control group, with an impressive ORR of 86.8% and a bone marrow uMRD rate of 51.9% [97]. The median participant age of 71 underscores the therapy’s potential effectiveness in an older population. However, the I+V regimen was associated with notable toxicity, with neutropenia as the most frequent grade 3 or higher adverse event. Notably, four cardiac or sudden deaths were reported in the ibrutinib–venetoclax treatment arm, raising concerns about cardiac toxicity [97]. In the light of this, FDA has not approved this combination of therapy. The I+V regimen has only been approved for CLL treatment by the European Medicines Agency (EMA) [106]. The next-generation BTKi acalabrutinib has demonstrated comparable PFS with reduced cardiac toxicity [107]. To further evaluate its potential, a phase III trial comparing acalabrutinib plus venetoclax versus venetoclax plus obinutuzumab in treatment-naïve CLL patients is currently underway (NCT05057494).

Building on the promising results of the CD20 inhibitor–venetoclax and ibrutinib–venetoclax combinations, the CLL2-BAG study (NCT02401503) explores the combination of venetoclax and obinutuzumab following bendamustine debulking in both treatment-naive and relapsed/refractory patients. This sequential approach achieved high rates of uMRD in peripheral blood (87%) and impressive ORR (95%) in both patient cohorts. These findings indicate that debulking with bendamustine can effectively reduce tumor burden, facilitating venetoclax-based treatment initiation [90]. Recent data suggested obinutuzumab triggers the destruction of lysosome and release of cathepsin D when used synergistically with venetoclax, leading to efficient killing of CLL cells [108]. The VENICE-1 trial further demonstrated high efficacy of venetoclax in relapsed or refractory CLL patients, including those who had previously received B-cell receptor inhibitors (BCRi) and those who had not [99]. These trials underscore the therapeutic value of CD20 inhibitor–venetoclax and BCRi–venetoclax combinations, establishing venetoclax as a critical option, especially for relapsed or refractory CLL.

Following this, the pivotal MURANO study (NCT02005471) compared venetoclax–rituximab (VenR) with bendamustine–rituximab (BR) in relapsed/refractory CLL patients, marking the first trial to directly compare conventional chemotherapy with a novel agent-based regimen. VenR treatment significantly improved PFS (84.9% vs. 36.3%) and MRD negativity rates (62.4% vs. 13.3%) over BR. Additionally, the VenR group showed a higher overall response rate (92.3% vs. 72.3%) and higher CR/CRi rates (26.8% vs. 8.2%). This phase III trial established the 2-year fixed-duration VenR as a new standard of care for relapsed/refractory CLL [93]. The superiority of venetoclax-based combinations over chemotherapy was further supported by the GAIA–CLL13 trial, where venetoclax–obinutuzumab, with or without ibrutinib, outperformed traditional chemoimmunotherapy in achieving uMRD and prolonging PFS. At month 15, the uMRD rate was notably higher in the venetoclax–obinutuzumab–ibrutinib group (92.2%) and venetoclax–obinutuzumab group (86.5%) compared to chemoimmunotherapy (52%). Three-year PFS rates were also superior for the venetoclax-based regimens, with 90.5% in the venetoclax–obinutuzumab–ibrutinib group, highlighting the benefit of deeper remissions [94]. The recently published 6-year results from the phase 3 CLL14 study further underscore the substantial long-term advantages of venetoclax–obinutuzumab (Ven–Obi) for untreated CLL patients compared to chlorambucil–obinutuzumab (Clb–Obi). Ven–Obi achieved a median progression-free survival (PFS) of 76.2 months versus 36.4 months for Clb–Obi and a 6-year OS rate of 78.7% compared to 69.2% [109]. Ven–Obi also extended time-to-next-treatment and showed a higher rate of uMRD five years post-treatment (7.9% vs. 1.9%), supporting it as an effective one-year fixed-duration therapy for CLL [109].

The aforementioned trials have positioned time-limited targeted therapies as the preferred frontline approach over traditional chemotherapy for CLL. For example, a fixed 1-year course of venetoclax and obinutuzumab is recommended for treatment-naïve CLL based on CLL14 trial, while a 2-year regimen of venetoclax and rituximab is used for relapsed or refractory cases. Time-limited treatments offer attractive benefits of deep remissions, frequently reaching uMRD, which enables patients to enjoy extended treatment-free periods. This strategy also reduces prolonged drug exposure, thereby minimizing cumulative side effects. For example, fixed-duration combination treatment with ibrutinib plus venetoclax may mitigate the development of resistance mechanisms associated with continuous single-agent targeted therapies and allow for effective retreatment with ibrutinib or ibrutinib plus venetoclax, thereby extending clinical benefit with these agents [110]. Yet, the primary drawback of time-limited therapy is the potential for disease relapse after treatment discontinuation, especially in patients with high-risk genomic profiles such as *TP53* mutations [109]. In contrast, continuous therapies, most commonly with BTKis like ibrutinib, are particularly beneficial for patients with high-risk genetic features, including *TP53* mutations and unmutated *IGHV*, who often relapse sooner on time-limited therapies [111]. Despite these advantages, long-term continuous therapy of ibrutinib can lead to cumulative toxicities, such as cardiovascular issues [112], as well as ongoing healthcare costs and logistical challenges, as patients must adhere to daily medication over extended periods [113].

The expanding role of MRD, despite its lack of standardization, offers a valuable measure for assessing and monitoring treatment response, supporting clinical decision-making and potentially help tailor treatment duration. Several trials, including the CAPTIVATE trial (NCT02910583), have explored MRD-guided therapy. In this trial, patients who achieved undetectable MRD were randomized to either placebo or continued ibrutinib, with results indicating that MRD-guided fixed-duration therapy could be feasible, as there was no significant difference in one-year disease-free survival between the two groups [95]. Moreover, MRD-directed treatment with ibrutinib combined with venetoclax has been shown to significantly improve PFS compared to the traditional FCR (fludarabine, cyclophosphamide, and rituximab) regimen in patients with naive CLL (FLAIR trial). OS outcomes also favored the ibrutinib–venetoclax combination, suggesting its potential as a superior therapeutic strategy in this patient population [100].

While venetoclax has significantly transformed the treatment landscape for chronic lymphocytic leukemia (CLL), its application is limited by hematologic toxicities and the risk of tumor lysis syndrome, often necessitating dose adjustments. This highlights the need for additional BCL-2 inhibitors, such as lisaftoclax [114] and sonrotoclax [115], which are being developed to address venetoclax-resistant CLL. Lisaftoclax was designed through computational modeling and selectively binds to BCL-2 (Ki < 0.1 nmol/L), disrupting BCL-2 complexes and demonstrating potent antitumor activity in CLL patients’ sample and preclinical models, including ibrutinib-relapsed malignancies [114]. Early phase 1 trials have demonstrated good tolerability, with 4 out of 22 evaluable patients with relapsed/refractory CLL achieving partial responses and an objective response rate of 63.6% [116].

Similarly, sonrotoclax, another second-generation BCL-2 inhibitor, has shown high efficacy against venetoclax-resistant *BCL-2* mutations, including the G101V mutation frequently observed in CLL patients who relapse after venetoclax therapy. Sonrotoclax’s unique binding mode within the BCL-2 pocket enables it to maintain robust activity in both cell lines and animal models, even when venetoclax loses effectiveness due to the G101V mutation [115]. An ongoing phase 1/2 study of sonrotoclax combined with zanubrutinib, a next-generation BTK inhibitor, has demonstrated favorable tolerability [117].

In addition to BCL-2 inhibitor (BCLi)-based combination therapies, several other treatment approaches have been developed. Immunotherapeutic strategies, including bispecific antibodies (such as epcoritamab and glofitamab) and CAR-T cell therapies targeting CD19 and CD20, have shown promise in treating relapsed/refractory CLL. Notably, the CD3-CD20 T cell engager epcoritamab is being tested in combination with venetoclax in a prospective phase I/II trial involving patients with relapsed/refractory CLL or SLL [118,119]. The phase I portion aims to establish the recommended dose level of epcoritamab for the subsequent phase II trial (NCT04623541) [120]. Additionally, a phase I/II clinical trial has demonstrated the potential of phosphatidylinositol-3-kinase (PI3K) inhibitors, such as umbralisib, and the anti-CD20 antibody ublituximab, particularly for CLL patients who are refractory to ibrutinib [121]. Furthermore, recent studies highlight the potential of the lysosomotropic agent siramesine, which, in combination with venetoclax, enhances CLL cell killing through reactive oxygen species (ROS) and a cathepsin-dependent mechanism [108].

## 7. Conclusions

CLL presents several persistent challenges in management and research due to its complex and heterogeneous nature. The disease is marked by substantial biological variability among patients, resulting in differences in disease progression, treatment response, and clinical outcomes. The advent of venetoclax has transformed CLL treatment and introduced new possibilities for therapy. Ongoing research efforts have focused on optimizing venetoclax-based combination regimens, informed by a growing understanding of the factors influencing both responsiveness and resistance to venetoclax.

## Figures and Tables

**Table 1:** Overview of mechanisms underlying resistance to venetoclax-based combination therapies.

Categories	Mechanism	Time to First Detection	Prevalence	Mechanisms of Resistance
Acquired genomic mutation	*BCL-2* G101V mutation [57,84]	19–42 months	10.4–46.7% (7/67–7/15)	Reduces venetoclax binding affinity
	*BCL-2* D103Y mutation [60,84]	23–39 months	11.9–16.7% (8/67–1/6)	Reduces venetoclax binding affinity
	*BTG1* mutation [61]	10.6–18.1 months	25% (2/8)	Impairs normal cell cycle regulation
	*CDKN2A/B* deletion [61]	4.5–14.5 months	37.5% (3/8)	Impairs normal cell cycle regulation
	*BRAF* mutation [61]	4.5 months	12.5% (1/8)	Increases expression of anti-apoptotic proteins like MCL-1
	*CD274 (PD-L1)* amplification [61]	21.8 months	12.5% (1/8)	Enhances immune evasion
	*TP53* mutations/deletions [85]	Not available (NA)	10–37%	Fails to initiate the apoptotic pathway
	Loss of 8p [86]	NA	36% (4/11)	Downregulates TRAIL-R signaling and upregulates WNT5A signaling pathways
	Gain of 1q [86]	NA	18% (2/11)	Upregulates MCL-1
	Loss of *PTEN* (loss of 10q) [86]	NA	18% (2/11)	Activates AKT pathway and subsequently upregulates BCL-XL
	*BTK* C481S mutation [86]	7.6–11.7 months	80.6% (37/46)	Confers resistance to BTK inhibitors, impacting combination therapies
	*PLCG2* mutation [86]	7.6–11.7 months	19.6% (9/36)	Confers resistance to BTK inhibitors, impacting combination therapies
Epigenetic changes in BCL-2	CpG island methylation in PUMA promoter [58]	NA	NA	Silences the PUMA gene and prevents apoptosis
Alteration of anti-apoptotic proteins and pro-apoptotic proteins	Increased expression of MCL-1 [64]	NA	NA	Allows evasion of apoptosis in response to BCL2i
	Increased expression of BCL-XL [64]	NA	NA	Allows evasion of apoptosis in response to BCL2i
	Increased expression of BFL-1 [64]	NA	NA	Allows evasion of apoptosis in response to BCL2i
	*BAX* mutation [65]	21–93 months	31.7% (13/41)	Fails to initiate apoptotic cascade
Metabolic reprogramming	Lymph node microenvironment [69]	NA	NA	Increases BCL-XL and MCL-1 and suppresses NOXA
	Increased OXPHOS [70]	NA	NA	Increases MCL-1 expression and activates PKA/AMPK pathway
	Loss of PUMA [58]	NA	NA	Enhances OXPHOS and ATP production
Aberrant signaling pathways	PI3K/AKT/mTOR pathway activation [73]	NA	NA	Increases levels of MCL-1 and BCL-XL
	NF-κB pathways activation [74]	NA	NA	Increases BCL-XL expression
	non-canonical NF-κB signaling activation [79]	NA	NA	Enhances expression of anti-apoptotic proteins
	ROR1 pathway activation [81]	NA	NA	Increases BCL-XL expression
	JAK1/2-STAT3 pathway [82]	NA	NA	Increases expression of BCL-2, MCL-1, and BCL-XL

**Table 2:** Summary of prominent venetoclax-based clinical trials in CLL.

Year	NCT	Study Name	Phase	Population	Intervention	n	ORR %	CR/Cri %	BM uMRD %	PB uMRD %	PFS %	OS %	Neutropenia/Anemia/Thrombocytopenia/Infection %
2018	02401503	CLL2-BAG [90]	2	Treatment-naïve vs. R/R	Bendamustine Obinutuzumab Venetoclax	35 31	100 90	8 ^#^	13 ^#^	91 83	100 83	100 90	44/11/12/14 ^#^
2019	02756897	MDACC [91]	2	Treatment-naïve	Ibrutinib Venetoclax	80	NR	88	61	NR	98	99	48/NR/2/5
2019	NR	CLARITY [92]	2	R/R	Ibrutinib Venetoclax	54	89	51	36	53	NR	100	66/NR/26/17
2018	02005471	MURANO [93]	3	R/R	Venetoclax Rituximab vs. Bendamustine Rituximab	194 195	93.3 67.7	26.8 8.2	27.3 1.5	83.5 23.1	84.9 36.3	91.9 86.6	60.8/15.5/13.4/17.5 44.1/22.9/22.3/21.8
2021	02242942	CLL14 [94]	3	Treatment-naïve	Venetoclax obinutuzumab vs. Chlorambucil Obinutuzumab	216 216	84.7 71.3	49.5 23.1	56.9 17.1	75.5 35.2	88.2 64.1	91.8 93.3	52.8/10.7/14.2/17.5 15.0/14.8/12.6/15.0
2021	02910583	CAPTIVATE [95]	2	Treatment-naive	Venetoclax Ibrutinib	159	96	55	60	77	95	98	42/NR/NR/67
2021	03824483	MSKCC [96]	2	Treatment-naïve	Zanubrutinib Obinutuzumab Venetoclax	39	100	57	89	89	NR	100	18/41/59/8
2022	03462719	GLOW [97]	3	Treatment-naïve	Ibrutinib Venetoclax vs. Chlorambucil Obinutuzumab	106 105	86.8 84.8	38.7 11.4	55.7 21.0	53.7 39.0	84.4 44.1	89.6 88.6	34.9/NR/5.7/15.1 49.5/NR/20.0/10.5
2023	02950051	CLL13 [98]	3	Treatment-naïve	FCR or BR vs. Venetoclax Rituximab vs. Venetoclax Obinutuzumab vs. Venetoclax Obinutuzumab Ibrutinib	229 237 229 231	75.5 NR 87.7 90.5	31.0 49.4 56.8 61.9	37.1 43.0 72.5 77.9	52.0 57.0 86.5 92.2	75.5 80.8 87.7 90.5	95.0 96.5 96.3 95.3	29.3/NR/NR/18.5 29.6/NR/NR/10.5 29.8/NR/NR/13.2 27.7/NR/NR/21.2
2024	02756611	VENICE-1 [99]	3b	BCRi-naïve R/R vs. BCRi-pretreated R/R	Venetoclax	191 67	85 64	35 27	27 ^#^	40 ^#^	NR NR	75 61	43/13/13/63 ^#^
2024	NR	FLAIR [100]	3	Treatment-naïve	Ibrutinib Venetoclax vs. FCR	260 263	86.5 76.4	59.2 49.0	65.9 49.8	92.7 67.9	97.2 76.8	98.0 93.0	10.3/0.8/2.0/21.5 47.3/15.5/10.0/17.4

^#^ Reported as total patient population; BCRi: B-cell receptor inhibitor; FCR: fludarabine–cyclophosphamide–rituximab; BR: bendamustine–rituximab; uMRD: undetectable minimal residual disease; ORR: overall response rate; CR/Cri: complete remission/complete remission with incomplete marrow recovery; PFS: progression-free survival; OS: overall survival; R/R: relapsed or refractory; NR: not reported.

## Data Availability

Not applicable.
